# Detecting rare earth elements using EnMAP hyperspectral satellite data: a case study from Mountain Pass, California

**DOI:** 10.1038/s41598-024-71395-2

**Published:** 2024-09-05

**Authors:** Saeid Asadzadeh, Nicole Koellner, Sabine Chabrillat

**Affiliations:** 1 Section of Remote Sensing and Geoinformatics, Helmholtz Centre Potsdam, GFZ German Research Centre for Geosciences, 14473 Potsdam, Germany; 2 Institute of Soil Science, Leibniz University Hannover, 30419 Hannover, Germany

**Keywords:** Neodymium, Bastnaesite, Carbonatite, Absorption feature analysis, Remote sensing, REE exploration, Mineralogy, Economic geology

## Abstract

Rare earth elements (REEs) exhibit diagnostic absorption features in the visible-near infrared region, enabling their detection and identification via spectroscopic methods. Satellite-based remote sensing mapping of REEs, however, has not been attainable so far due to the necessity for high-quality hyperspectral data to resolve their narrow absorption features. This research leverages EnMAP hyperspectral satellite data to map REEs in Mountain Pass, California—a mining area known to host bastnaesite-Ce ore in sövite and beforsite carbonatites. By employing a polynomial fitting technique to characterize the diagnostic absorption features of Neodymium (Nd) at ∼740 and ∼800 nm, the surface occurrence of Nd was successfully mapped at a 30m pixel resolution. The relative abundance of Nd was represented using the continuum-removed area of the 800 nm feature. The resulting map, highlighting hundreds of anomalous pixels, was validated through laboratory spectroscopy, surface geology, and high-resolution satellite imagery. This study marks a major advancement in REE exploration, demonstrating for the first time, the possibility of directly detecting Nd in geologic environments using the EnMAP hyperspectral satellite data. This capability can offer a fast and cost-effective method for screening Earth’s surfaces for REE signature, complementing the existing exploration portfolio and facilitating the discovery of new resources.

## Introduction

Rare Earth Elements (REEs) are essential to many modern technologies, including electric vehicles, wind turbines, and smartphones^1–3^. This broad range of applications, combined with increasing demands and disruptions in the supply chain, has transformed REEs into strategic commodities and critical raw materials^4^. REEs are a group of metallic elements with similar chemical properties comprising the lanthanides (atomic number 57 to 71) plus Y (39), commonly divided into light (LREE) and heavy (HREE) subgroups, comprising La to Eu and Gd to Lu + Y, respectively^5^.

REEs naturally occur in a diverse array of minerals, including carbonates, phosphates, silicates, and oxides of which carbonates and phosphates constitute the most abundant and economically valuable minerals^2^. The REE carbonates include the fluorocarbonate minerals bastnaesite, synchysite, and parisite^6^. The RE_2_O_3_ content of these minerals is exceptionally high, reaching up to 75 wt.% in bastnaesite^7^. LREEs typically concentrate in carbonates (i.e., bastnaesite; (Ce,La)(CO_3_)F) and phosphates (i.e., monazite; (Ce,La,Nd,Th)PO_4_), whereas HREEs are commonly hosted by oxides and, partly, by phosphates, including xenotime ((HREE,Y)PO_4_). Due to chemical similarity (ionic radii and oxidation states), REEs often substitute for one another and co-occur within the same mineral species^5^.

Contrary to their name, REEs are relatively abundant in the Earth's crust, though economically viable deposits are uncommon. Several deposit classes are recognized to host REEs^2^, with carbonatites being the predominant sources, accounting for more than 70% of global REO (Rare Earth Oxides) production. Two notable examples of such deposits are the Bayan Obo mine in China and the Mountain Pass in the US^8^. Carbonatites are defined as rocks with > 50% primary magmatic carbonates. Geologically, they occur in continental settings and based on their mineralogy and petrographic texture are divided into three distinct classes: calcitic (also referred to as sövite), dolomitic (beforsite), and ankeritic (ferrocarbonatite)^2,5^. Carbonatites predominantly host LREEs such as La, Ce, Pr, and Nd, with bastnaesite being the primary mineral exploited in many related deposits^5,7^.

**Fig. 1:**
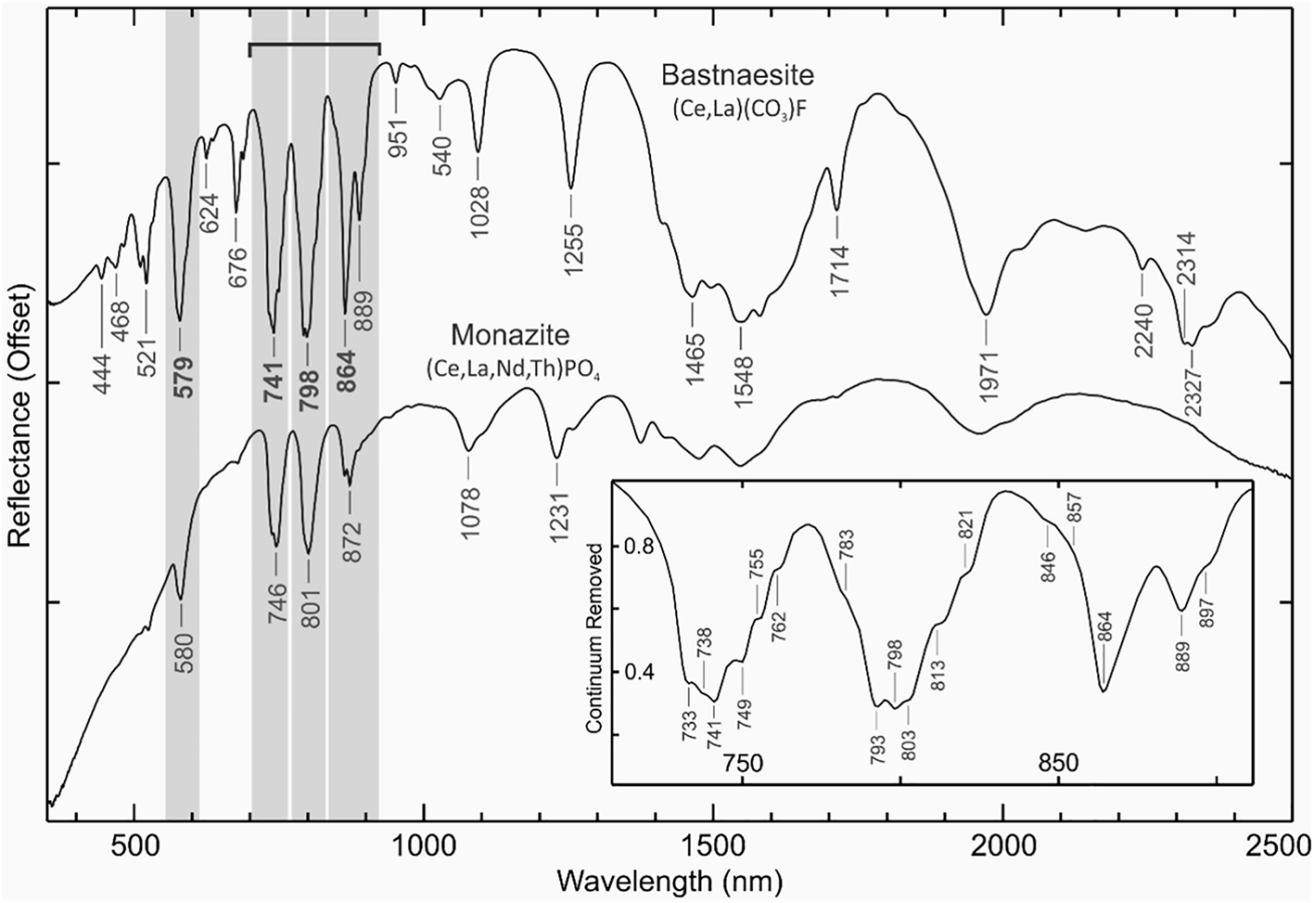
Spectral signature of the rare-earth mineral bastnaesite in the VNIR–SWIR range. The spectrum of monazite is shown for comparison—data sourced from the USGS spectral library^21^. The key absorption features of Nd in the VNIR range are bolded. The inset graph provides a closer view of bastnaesite’s absorbing bands between 700 and 910 nm (marked by the solid bar), covering three diagnostic absorption features. The gray columns represent the spectral ranges used for polynomial fitting and remote sensing mapping of Nd in the study area.

The technique of reflectance spectroscopy has recently emerged as a fast and cost-effective analytic tool for detecting and quantifying REEs. Several REEs, including Nd^3+^, Pr^3+^, Sm^3+^, Dy^3+^, Er^3+^, Ho^3+^, and potentially Eu^3+^ and Tm^3+^ exhibit diagnostic absorption features in the visible-near-infrared (VNIR; 400–1000 nm) and partly in the shortwave-infrared (SWIR; 1000–2500 nm) wavelengths, allowing them to be detected via spectroscopic methods^6,9–16^. The narrow absorption bands of REEs observed in the VNIR, as exemplified in Fig. 1, are attributed to 4f-4f intra-configurational electron transitions^6,11,15^.

Despite the effective shielding of the 4f orbitals by the 5s and 5p closed shells, the corresponding energy levels in the mineralogic phases are not fixed and rather undergo subtle changes, depending on the ligand type, coordination number, and polyhedron asymmetry. This variability leads to shifts in the position of absorbing bands within mineral phases, typically on the order of ∼10 nm in the VNIR range^6,15,17^. In other words, while the absorbing bands arise from REE ions, the host mineralogy plays an important role in determining the exact position of the absorption features and their intensities. The spectral behaviors of rare-earth minerals are already cataloged in several specialized spectral libraries^6,18–21^.

A growing number of studies have shown that Nd is the most spectrally active and readily detectable REE via spectroscopic methods^9,12,14,22^. The identification of Nd typically relies on the characterization of its most prominent and defining absorption features at ∼580, ∼740, ∼800, and ∼865 nm (see Fig. 1). By leveraging these distinctive features, the hyperspectral imaging technology has been able to detect Nd across various scales and conditions, spanning from close-range scanning of thin sections^23^ and hand specimens in laboratory settings^6,24^ to the mapping of vertical outcrops on the ground^25,26^ and open-pit mines from airborne platforms^27,28^. More recently, Unmanned Aerial Vehicle (UAV)-based imaging systems have been employed to map REE-rich veins and outcrops at very high spatial resolutions^29^.

In contrast, direct detection of REEs by spaceborne satellite systems, such as ASTER and WorldView-3 multispectral instruments, has been unachievable, mainly due to the coarse spectral resolutions of multispectral datasets, rendering them unable to resolve the sharp yet narrow absorption features of REEs (Fig. 1), regardless of their spatial resolution^28,30^. While previous laboratory-based spectral simulations have demonstrated the potential of hyperspectral instruments, including the EnMAP satellite system, for direct REE detection^9^, the capability of the corresponding dataset has remained untested in real-world conditions.

This paper aims to bridge this gap and pave the way for further research by analyzing the EnMAP imaging spectroscopic data collected over the Mountain Pass REE mine in California, USA. Our study aims to prove the concept and recognize the potentials and limitations of spaceborne hyperspectral datasets for the direct detection and mapping of REEs. This is accomplished by studying the well-exposed, high-grade REE mine of the Mountain Pass area using the EnMAP imaging data at 30 m spatial resolution. Furthermore, the study seeks to evaluate the effectiveness of spectroscopic-based processing methods for REE detection aiming to provide a reliable site-independent mapping technique applicable to EnMAP and analogous hyperspectral remote sensing datasets.

## Geology of the Mountain Pass area

Mountain Pass is located in southeastern California, approximately 65 km southwest of Las Vegas, in the Mojave Desert. Geologically, the area comprises a collection of Mesoproterozoic alkaline silicate intrusions (ca. 1.41 Ga) ranging in composition from mafic (shonkinite) through syenite to alkali granite. This suite is associated with a series of contemporaneous carbonatite dikes and intrusions^31^. The northern and eastern parts of the area consist of Proterozoic schists and gneisses, granitoids, and minor carbonatite intrusions. In contrast, the south and southeast are characterized by Paleozoic limestone, dolostone, and sandstone intruded by Jurassic granitic rocks and Cretaceous granodiorites^28^. The central and western parts are covered by folded, thrust-faulted Paleozoic carbonate and quartzose rocks^28^. A more detailed description of the area’s geology can be found in Castor^32^, Mars^30^, Mariano and Mariano^33^, and Watts, et al.^31^.

The carbonatites and the associated alkaline plutons constitute a suite of roughly tabular to lenticular, moderately west-dipping intrusions trending north-northwest within the ultrapotassic intrusive rocks^32^. The largest body, known as the Sulphide Queen carbonatite, is located in the center of the area, measuring 700 m in width and up to 150 m in thickness^10^ and hosting the largest REE deposit in the US^7,31^. The Sulphide Queen carbonatite primarily consists of bastnaesite-barite sövite (calcitic) and bastnaesite-barite-dolomite (beforsite), or a mixture of both (dolomitic sövite), with the dolomitic carbonatite being more prevalent^32^.

Although the size of the Sulphide Queen carbonatite is modest, the orebody is highly enriched in LREE. The ore, which is recognized to be of igneous origin, typically contains 10–15% bastnaesite-Ce, 65% calcite/dolomite, and 20–25% barite. The bastnaesite mineral crystals are coarse-grained, typically measuring 300 µm in diameter with an average REE composition of 45.50% Ce, 15.82% Nd, 4.65% Pr, and 1.83% Sm, with lower quantities of Eu, Gd, Dy, Ho, and Er^32^. Other LREE-bearing accessory minerals are parisite, synchysite, monazite, and, less often, allanite^33^. Mining activities in the Sulphide Queen stock started in 1952 and ceased by 2002, leaving a reserve of > 20 million metric tons of ore at an average grade of 8.9% REO in place^34^. By 2007, the extraction of selected REE commodities from stockpiles resumed, and since 2018, the mine has been reactivated in response to the increased demand for REEs and geopolitical forces^31^.

Besides the main carbonatite body, there are numerous steeply inclined carbonatite dikes in the area, with the majority occurring in the vicinity of the Sulphide Queen orebody. Several of these dikes, particularly those adjacent to the mine, are known to contain bastnaesite, although most have low REO contents. A fenitized zone approximately 4 km southeast of the mine is also reported to host REE prospects containing allanite and bastnaesite minerals^32^.

The target area has been the focus of several remote sensing studies, primarily aimed at lithologic mapping using different multi- and hyperspectral datasets^28,30,35^. Based on field observations, vegetation covers between 10 to 30% of the surface, making it suitable for remote sensing studies.

## Results

**Fig. 2:**
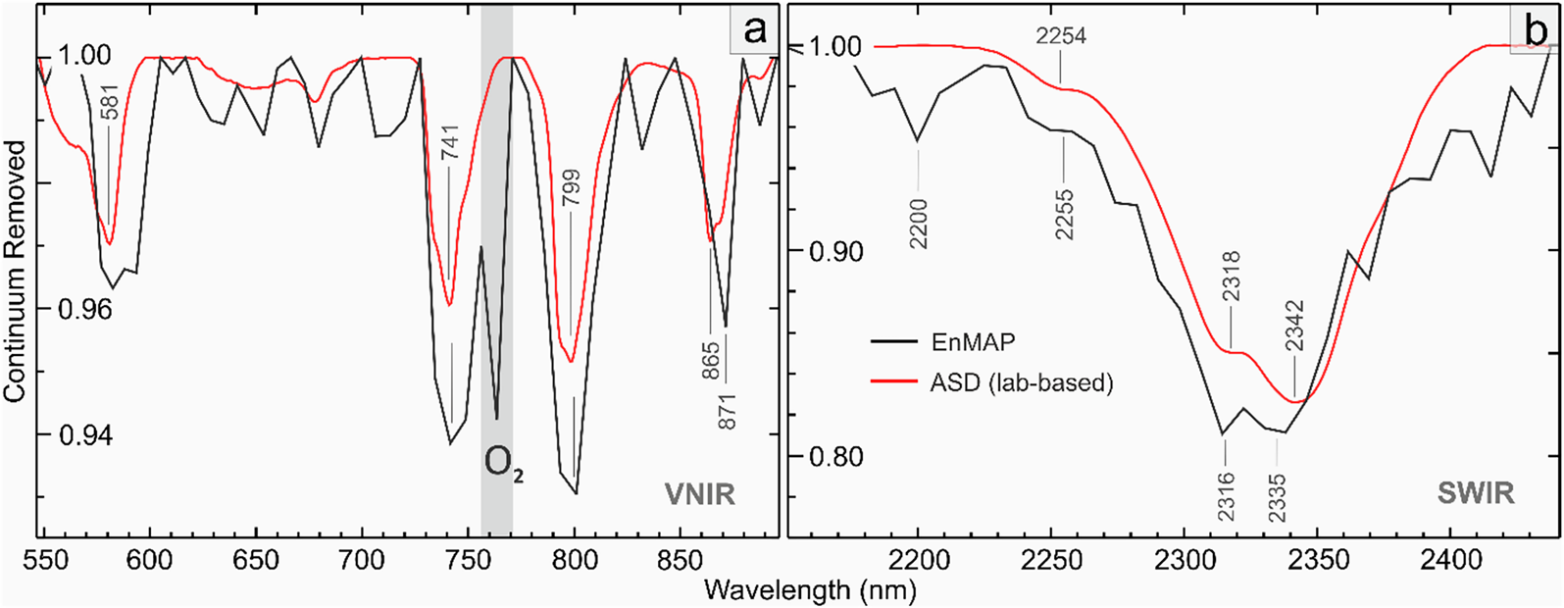
Continuum-removed reflectance spectra from EnMAP (in black) over the Sulphide Queen Mine compared to the laboratory-based spectrum of bastnaesite-rich ore from the mine site. The spectra are plotted in the VNIR (**a**) and SWIR (**b**) spectral ranges in native resolution. The laboratory spectrum, acquired using an ASD spectrometer, is sourced from the datasets published by Neave, et al.^9^. The vertical gray column in (**a**) highlights the EnMAP band affected by oxygen’s residual absorption feature at 760 nm. The minimum wavelengths were calculated by the polynomial fitting technique described in section "Processing methodology". Note that the two graphs have different Y-axis scales.

The EnMAP data successfully resolved the REE- and carbonate-related features in the VNIR and SWIR ranges (Fig. 2). In the VNIR, it identified four diagnostic absorption features at ∼580, ∼740, ∼800, and ∼870 nm (Fig. 2a). Within the SWIR range, it detected a deep carbonate feature at 2335 nm and two characteristics features related to bastnaesite at 2255 and 2316 nm (Fig. 2b). The contained carbonate was identified as calcium carbonate, distinguished by a pronounced absorption feature at 2335 nm and the absence of a ferrous iron feature in the VNIR.

The representative pixel spectrum over the open-pit mine shows good agreement with the laboratory spectroscopy of the orebody. Both datasets exhibit a comparable spectral pattern with the same number of absorption features and intensities across the VNIR and SWIR ranges, following continuum removal (Fig. 2). Notably, the positions of absorption minimums for the 580, 740, and 800 nm features are nearly identical in both datasets and for the characteristic features of bastnaesite, the difference is in the order of 1–2 nm (2255 vs. 2254 nm and 2216 vs. 2218 nm). However, the minimum wavelength differences for the 870 and 2330 nm features are significant. In the EnMAP data, the Nd feature occurs at a slightly longer wavelength (871 vs. 865 nm), and the carbonate feature appears at a shorter wavelength (2335 vs. 2342 nm) (Fig. 2). The latter is likely due to the spectral mixture of calcic carbonate with bastnaesite absorption features at the EnMAP ground sampling distance of 30 m. It is noteworthy that the bastnaesite spectrum depicted in Fig. 1 exhibits a different pattern compared to the laboratory plot in Fig. 2b. The features at 2254, 2318, and 2342 nm appear respectively at 2249, 2314, and 2327 nm in Fig. 1, possibly due to the complex/mixed mineralogy of the sample from the Mountain Pass. The 2255 nm feature observed in Fig. 2b is speculated to arise from the hydroxyl bond in bastnaesite^6^.

Further distinctions include variations in the width of absorption features, which tend to be broader in the EnMAP data. Additionally, the right side of the 740 nm absorption feature in the EnMAP data is affected by a widespread residual O_2_ absorption feature (Fig. 2a). EnMAP also resolves an additional feature at 2200 nm, likely linked to clay minerals (Fig. 2b). It is worth noting that the laboratory spectrum of this study closely resembles the spectral plot (published in Mars^30^ i.e., Spectrum A in Fig. 7).

**Fig. 3:**
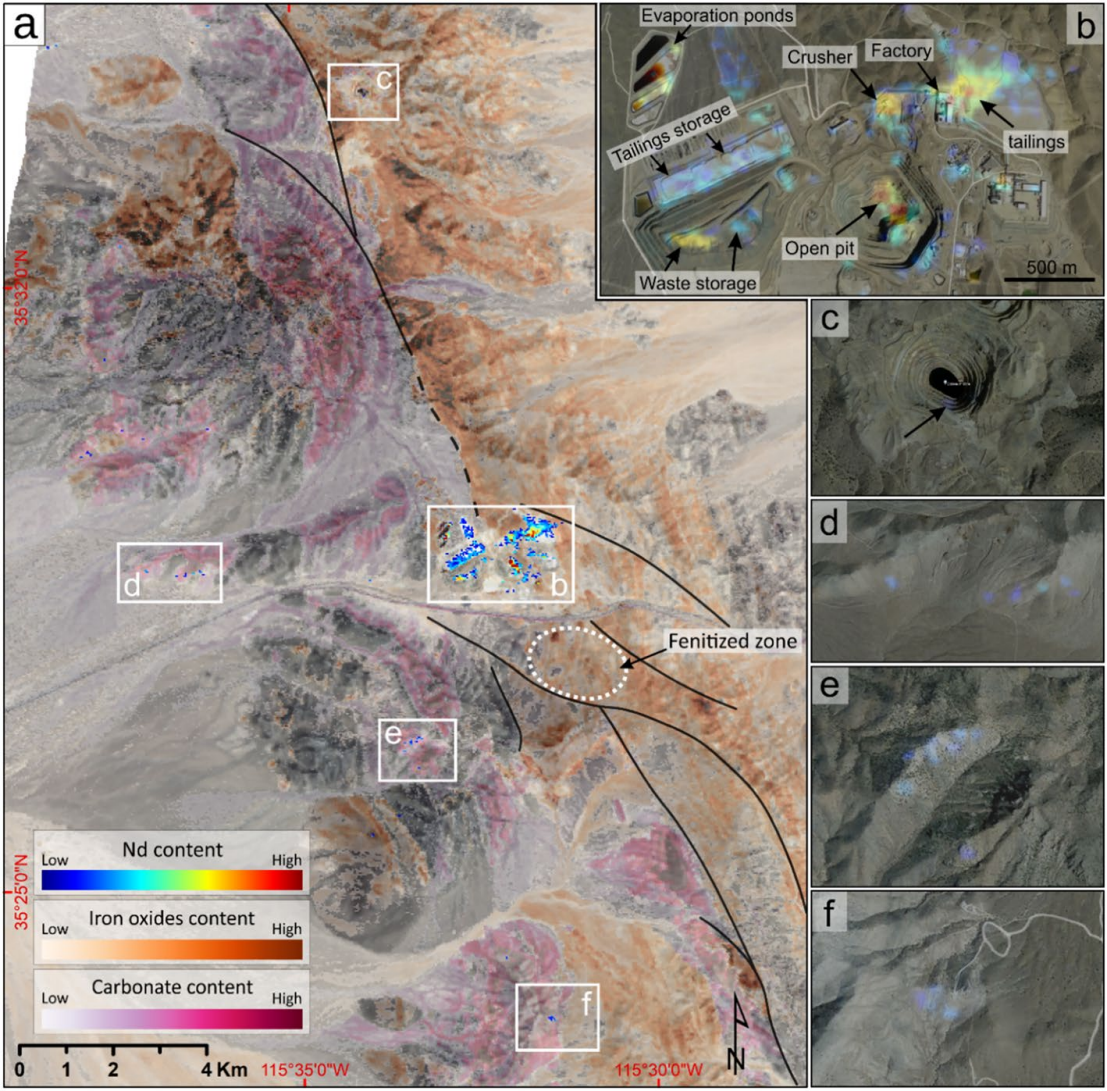
The spatial distribution and relative abundance of REEs in the Mountain Pass area, California. (**a**) Nd anomaly map (blue-red) yielded from spectral analysis of EnMAP hyperspectral data overlaid on enhanced albedo imagery. The area of the 800 nm absorption feature is used to indicate the relative abundance of Nd in the mapped pixels. The relative abundance of iron oxide and carbonate minerals are depicted in the background by orange and purple-red colors, respectively. (**b**–**f**) The same Nd anomalies from (**a**) overlaid on high-resolution satellite imagery of the area available on Google Earth. The data are from 29^th^ March 2021 at a ground sampling distance of ∼1 m. White rectangles in (**a**) define the outline of the images shown in (**b**) to (**f**). Major faults are shown by solid/dashed black lines.

The distribution and relative abundance of Nd across the Mountain Pass area is illustrated in Fig. 3a. Here, the spectral signature of Nd was mapped not only over the open-pit mine but also over the stockpiles, tailings storages, evaporation ponds, the crusher site, and the concentrator facilities (Fig. 3b). The anomalies detected over the concentrator facility are probably the result of REE-bearing dust being transported westward from the mine crusher by the prevailing wind direction in the Mojave Desert. The Nd signature was also detected in several localities beyond the mining site, including at the edge of the Colosseum mine northward (Fig. 3c) and over carbonate rocks in the west and southwest of the study area (Fig. 3d–f). However, unlike the anomalies observed in the mining area, which form clusters of connected pixels, the peripheral anomalies are generally limited to a few pixels. In total, 740 pixels encompassing an area of 880,000 m^2^ were identified to exhibit Nd features. The most prominent absorption feature was observed over the evaporation ponds, while the faintest was detected above the tailing storages (Fig. 3b). The mapping method detected no anomalies over the fenitized zone southeast of the mining area (Fig. 3a).

In Fig. 3a, the relative abundance of iron oxide minerals (i.e., hematite and goethite) and carbonates (i.e., calcite and dolomite) are depicted in orange and purple-red colors, respectively. Iron oxides are predominantly found in the NW to SE of the area, whereas carbonates are more abundant westward.

**Fig. 4:**
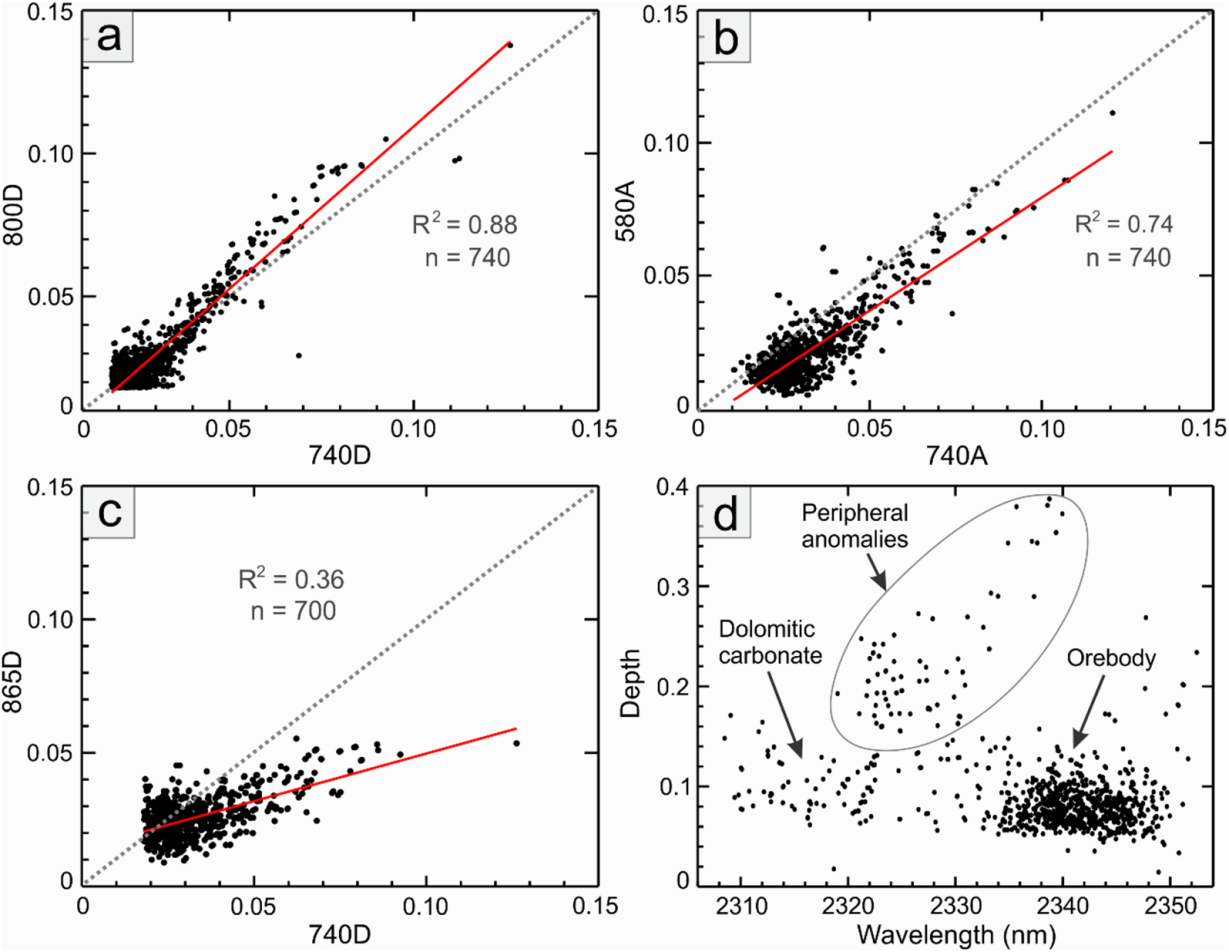
Scatterplots of the spectral parameters of the Nd-bearing pixels derived from EnMAP data over the Mountain Pass area. (**a**) plot of the absorption depth at ∼740 nm (740D) against 800D. (**b**) plot of the absorption area at ∼740 nm (740A) against 580A. (**c**) plot of the absorption depth at ∼740 nm (740D) against 865D. (**d**) Plot of the minimum wavelength of the carbonate absorption feature against its depth for the pixels containing REE absorption features. The plotted data corresponds to the Nd anomalies mapped in Fig. 3a. The solid red and dashed gray lines depict the best-fitted line to the data and the 1-to-1 line, respectively.

The statistical relationships between different spectral parameters within the mapped pixels are summarized in the scatterplots of Fig. 4. During the spectral processing, it was noted that the minimum wavelengths of the 740 and 800 nm features vary within the ranges of 735–755 and 793–805 nm, respectively. These features exhibit a strong correlation in terms of absorption depth (R^2^ = 0.88; Fig. 4a), with the 800 nm feature appearing to be slightly deeper (see also Fig. 2a). The interfering effect of residual O_2_ absorption (Fig. 2a) seems to be largely mitigated after excluding the corresponding band from the calculations.

In contrast, the less prominent Nd feature at ∼580 nm (Fig. 2a), while visually discernible in several Nd-bearing pixels, was found unsuitable for Nd mapping. This is primarily due to significant interferences from other scene components comprising green vegetation, causing noticeable shifts in the feature’s minimum wavelength making it difficult to track the via processing method. Nevertheless, the area of this feature correlates well with the area of the 740 nm feature (R^2^ = 0.74; Fig. 4b) and the 800 nm feature (R^2^ = 0.66; not shown). The feature at ∼865 nm, although noticeable in some pixels over the orebody (Fig. 2a), was not well-developed and therefore not resolvable in the EnMAP data. Statistically, it shows a weak correlation (R^2^ = 0.36) with the depths of the absorption features at ∼740 and ∼800 nm (Fig. 4c).

Figure 4d depicts the plot of carbonate minimum wavelength against its depth for the pixels mapped in Fig. 3a. In this plot, pixels from over the mining area and orebody exhibit wavelengths ranging from 2335 to 2350 nm and a relatively shallow carbonate absorption, typical of bastnaesite-rich calcic carbonatite. Pixels with similar absorption depths but shorter wavelength ranges (2310 to 2330 nm) were interpreted to arise from REE-bearing dolomitic carbonatite. The third cluster in Fig. 4d represents isolated pixels mapped at the periphery of the mining area over carbonate rocks (highlighted in Fig. 3d–f). These pixels are characterized by very shallow features at ∼740 and ∼800 nm but a deeper carbonate feature at wavelengths ranges between 2320 to 2340 nm. Verifying the presence of REEs/Nd in these pixels would indeed require ground truthing.

## Discussion

For reliable detection of REEs using spectral remote sensing data, it is essential to resolve multiple absorption features within the dataset. While some studies have successfully used three and occasionally four of the diagnostic absorption features of Nd^23,24,36^, many others have shown that not all the distinctive absorption features in the VNIR range, particularly those at ∼580 and ∼870 nm (refer to Figs. 1 and 2a), are consistently present and resolvable in spectral data, even under optimal laboratory conditions^12,14,18,22,37^. Consequently, it is not surprising that the EnMAP data can only resolve the most prominent absorption features of Nd at ∼740 and ∼800 nm. This is consistent with the results of other remote sensing studies conducted to map REEs under open-air conditions using a UAV platform^29^. Conversely, relying solely on a single absorption feature can introduce large uncertainty in Nd detection^12^.

As demonstrated in this study, the minimum wavelength of the absorption features is as important and informative as the feature depth for REE detection. However, the minimum wavelengths of the absorption features are highly variable in spectral data. In laboratory studies, the minimum wavelengths of the 580, 740, and 800 nm features have been reported to vary from 575 to 590 nm, 740 to 747 nm, and 799 to 805 nm, respectively^9^. The variations retrieved from the EnMAP data, however, cover a wider range varying from 581 to 597 nm, 735 to 755 nm, and 793 to 805 nm, respectively. This wide range could be attributed to various factors, including intrinsic variations in the minimum wavelength of bastnaesite (typically on the order of ∼10 nm, as stated in the introduction), the co-occurrences of other REE-bearing minerals such as parisite, synchysite, and monazite inside the pixel footprint, the intimate/areal mixture of rare-earth minerals with other lithologic/background constituents (see below), the uncertainty of the retrieval method, and above all, limitations in the spectral sampling interval of EnMAP (i.e., 6.5 nm) compared to laboratory data.

It is important to note that each of these absorption features results from the superposition of several absorbing bands. For instance, the pronounced absorption feature at ∼740 nm is the result of at least six narrow absorbing bands centered at 733, 738, 741, 749, 755, and 762 nm (see the inset plot in Fig. 1), of which only four (i.e., at 734, 741, 747, and 757 nm) are discernible in the laboratory data of Fig. 2a. A thorough analysis of these features can help characterize the mineralogical state of REEs and potentially unravel the presence of other REEs beyond Nd in spectral data.

In general, the ability to detect REEs spectrally could be affected by the following factors:

(i) *The overall albedo of the target and the contrast of the REE host with its background constituents*. High proportions of opaque minerals such as magnetite (and allanite in non-carbonatite deposits) have been observed to dampen the spectral signal, contributing to low reflectance levels from the samples/surfaces and thus difficulty in REE detection^9,14^. In contrast, brighter backgrounds, exemplified here by the dominance of calcic carbonatite, can facilitate the detection of REEs.
(ii) *The relative proportion of ferric (Fe*^*3*+^*) iron minerals*. The broad and intense absorption features of iron oxide minerals (i.e., hematite and goethite) in the VNIR region are reported to suppress the REE features significantly^10,12,22,25^. Simulated experiments have shown that even 1 wt.% of iron oxides can attenuate REE-related features, with the 580 and 870 nm features being particularly susceptible to suppression. In the range of 2 to 5 wt.%, iron minerals can readily dampen the features arising from 0.5 wt.% Nd, and at the 10 wt.% level, the REE features disappear entirely due to the dominance of ferric iron absorptions in the VNIR range^13,22^. As a general rule, the two weaker absorptions at ∼580 and ∼870 nm are more vulnerable and often go undetected in many spectral measurements (Todd Hoefen, personal communication). In the Mountain Pass area, although iron oxides are scarce over the open-pit mine, they are prevalent in the surrounding area, particularly over the alkaline intrusions eastward of the major fault lines (Fig. 3a), contributing to the suppression of potential Nd features.
(iii) *The fraction of vegetation cover*. The presence of the green peak and chlorophyll absorption, respectively at ∼550 and ∼590 nm can undermine the REE feature at 580 nm. Presumably, the interference from vegetation in this area has impeded the mapping of the 580 nm feature in the EnMAP data, despite its existence and reasonable correlation with the 740 nm feature (Fig. 4b). This is supported by the observation that pixels with the highest incidence of false-positives when using only the 580 nm feature for Nd mapping, are spatially associated with the highest Normalized Difference Vegetation Index (NDVI) values calculated from the same data. The interference from chlorophyll absorption may also explain the shift in the minimum wavelength of the 580 nm feature towards longer wavelengths (581 to 597 nm in EnMAP vs 575 to 590 nm in laboratory data). Further studies are required to understand the sensitivity of REE features to vegetation coverage/fraction.
(iv) *The atmospheric correction effects*. As illustrated in Fig. 2a, the distinct O_2_-related absorption at 760 nm can interfere with the 740 nm feature of Nd. When the 740 nm feature surpasses the residual O_2_ absorption, excluding the corresponding band from calculations, as demonstrated in this study, offers a simple yet effective solution to the problem. However, in situations where the feature is weakly developed and oxygen’s residual absorption predominates, excluding the band may not resolve the issue and could potentially lead to miscalculations of the spectral parameters, affecting the Nd mapping results. In contrast to O_2_, the residual water vapor effect appears as noise beyond 890 nm suppressing the 870 nm feature of Nd. While it is likely that the 870 nm feature may not be well-developed in the first place, the impact of water vapor residuals in weakening this feature within the EnMAP data needs to be considered. A more robust atmospheric correction procedure could certainly lead to better retrieval of REE signatures from EnMAP data.
(v) *The grain size effect.* The size of REE-bearing grains is another factor affecting the intensity of Nd absorption features and, consequently, its detectability. Larger grain sizes absorb more light, leading to deeper absorption features^9^. In the Mountain Pass area, the relatively large bastnaesite grains, with an average diameter of 300 μm^32^, could be the reason behind the increased depth and width of absorption features in the EnMAP data (Fig. 2a). However, variability in Nd grade and the scale effect (30 m image pixel vs point-scale ASD data) may have also played a role in this behavior.
(vi) *The proportion of Nd (and total REEs)*. Since the intensity of absorption features is proportional to the concentration of Nd in a sample/pixel, a higher concentration results in more pronounced absorption features, thereby facilitating spectral detection^9,12,14,17^. Based on this premise, while the exceptionally high concentration of Nd in the Mountain Pass area appears to have facilitated the remote sensing mapping, it is noteworthy that Nd was also detected over the tailings and waste storage sites (Fig. 3b), indicating the detectability of lower grades of Nd via EnMAP data. In contrast, EnMAP was unsuccessful in mapping any Nd signatures over the fenitized zone and the adjacent areas (encircled in Fig. 3a). This can be attributed to the small size of the carbonatite veins in this zone, the low content of REEs (Nd), as reported by Castor^32^, and the prevalence of iron oxides (see Fig. 3a). Similarly, no carbonate signatures were detected over these veins using EnMAP's SWIR bands.
  It's important to note that the depth of Nd's absorption features is reportedly influenced by the Nd to ΣREE (total REE) proportion, with higher ratios resulting in more pronounced absorption features^29^. The smallest REE-bearing target detectable at the 30 m pixel size of EnMAP, as well as the lowest level of Nd detectable spectrally (corresponding to the detection limit of EnMAP data), is currently unknown and should be addressed in future studies considering the noted factors. However, since reflectance spectroscopy has demonstrated a relatively low detection limit for Nd, ranging from 1000 to < 200 ppm^9,10,12,29^, it can be expected that under optimal environmental conditions, the EnMAP instrument will be sensitive to low grades of Nd/REEs in a pixel (see below).
(vii) *The sensor effects.* While EnMAP data exhibits excellent quality in both the VNIR and SWIR ranges, it is acknowledged that the bands at the longer wavelength end of the VNIR detector display erratic nonlinear behavior due to the fringing effect (EnMAP's unpublished internal report). The challenges faced by EnMAP in resolving the 870 nm feature may, in part, be attributed to this phenomenon, particularly beyond 900 nm, where the right shoulder of the feature is located.
  Comparing the outcomes of this study with the analysis conducted by Mars^30^ using WorldView-3 data underscores the significance of spectral resolution over spatial resolution in mapping REEs. Because despite WorldView-3's exceptional spatial resolution, it could not map Nd occurrences in the area. In contrast, EnMAP, with a spatial resolution of 30 m, succeeded due to its high spectral resolution and calibration accuracy. Certainly, high spatial resolution hyperspectral data can enable the detection and mapping of meter-scale veins in geologic outcrops. However, for spaceborne remote sensing data with restrictions in spatial resolution, enhancing the SNR and spectral resolution can increase their sensitivity and utility for REEs.
(viii) * The spectral processing method.* After testing various spectral processing methods, which included multiple target detection algorithms, similarity measures, feature fitting algorithms, and a support vector machine classifier^38^, it was observed that the choice of processing method has implications for successful Nd detection. Remarkably, none of the tested methods were able to generate results comparable to the map shown in Fig. 3a (using the mapped pixels as endmembers/training data), highlighting the superiority of the absorption feature analysis and polynomial fitting technique for REE detection. This may explain why prior attempts to map REEs in the area using airborne data e.g.,^28,35^ were not very successful. The main advantage of the approach employed in this paper is that it does not require a priori knowledge about REE occurrences in a given area and rather it relies on the spectroscopic knowledge of rare-earth minerals for remote sensing mapping.

In summary, the ability to detect REEs using hyperspectral remote sensing data depends on geological and instrumental constraints. Geologically, it depends on the size of the target, its exposure level, the contained level of REEs, and the composition of accompanying minerals. Instrumentally, it primarily depends on the imaging system's SNR and spectral resolution, followed by spatial resolution, and the quality of atmospheric correction and processing methods.

While in this study, hundreds of pixels were identified to contain Nd, in similar remote sensing studies in the future, the detection of REE signatures, even in a single image pixel, should be considered promising for subsequent field studies. While identifying the rare-earth mineralogic host, as achieved here, may not be always practical or necessary for remote sensing studies, detecting the carbonate signature (via SWIR bands) in a carbonatite host^39,40^ could further support the presence of REE in a target. It is important to note that as a remote sensing method, our methodology can only detect REE signatures at the surface without the ability to penetrate to depth.

## Conclusion

This study demonstrated that EnMAP hyperspectral satellite data can directly and efficiently detect REEs in geological environments. EnMAP successfully resolved the distinctive absorption features of Nd at 740 and 800 nm arising from the Nd-rich bastnaesite ore in the Mountain Pass area. While EnMAP could resolve the feature at ∼580 nm, the feature was not suitable for REE mapping due to its low intensity and interference with iron oxides and the chlorophyll absorption feature occurring at ∼590 nm. EnMAP data was unable to confidently resolve the feature at ∼870 nm. The absorption feature analysis and polynomial fitting technique proved to be a superior and effective processing method for characterizing the prominent REE absorption features and mapping the occurrences and relative abundances of Nd in imaging spectroscopic data.

Detecting the spectral signature of REEs by spaceborne imaging spectroscopic data can take exploration activities for REEs to another level. Conventionally, carbonatite bodies, as the primary hosts of LREEs, have been explored through geophysical methods relying on airborne magnetic and radiometric surveys^41^. Introducing a remote sensing approach capable of detecting the contained REEs directly and mapping the underlying host mineralogy and alteration aureoles can complement the existing exploration portfolio, facilitating the discovery of new carbonatite bodies and REEs resources.

The EnMAP satellite data with its global coverage can be used to screen large areas for REE signatures. However, given its 30 m spatial resolution, it is expected to mainly detect well-exposed targets of sufficient Nd quantities/sizes in arid to semi-arid regions of the world. Advancements in atmospheric correction procedures and processing methods can aid in detecting lower grades and smaller Nd-bearing targets. Because REEs are often associated with each other, and because the host mineralogy does not highly modify the REE-related absorption features, remote sensing mapping of Nd should serve as an exploration pathfinder for light (and potentially heavy) REEs, irrespective of their deposit types.

Future work will involve establishing quantitative relationships between Nd grade and spectral signatures and testing the methodology across a diverse range of REE-rich deposits/prospects with varying levels of light/heavy REEs, outcrop exposures, geologic /conditions, and vegetation coverage. This could help to better understand the spectral behavior of REEs at EnMAP resolution and determine the instrument’s full capability in detecting and mapping REEs occurrences remotely.

## Materials and methods

### EnMAP hyperspectral data

The EnMAP (Environmental Mapping and Analysis Program) hyperspectral satellite system was launched into orbit on April 1, 2022, and since November 2022 has been in routine operation^42^. EnMAP is a German satellite mission designed and operated by the German Aerospace Center (DLR) and funded by the Federal Ministry for Economic Affairs and Climate Action (BMWK) of Germany^43,44^.

The EnMAP data of the study area, collected on July 7th, 2022 at 18:47:54.75 UTC (11:47 local time) was obtained from the EOWEB® portal. The data was ordered using the following settings: Level 2A data with ozone and terrain corrections enabled, with no spectral interpolation, resampled by the nearest neighbor method. The data was processed using the March 2023 version of the EnMAP processor. The Level 2A orthorectified surface reflectance data of EnMAP comprises 224 spectral bands at 30-m spatial resolution. The VNIR bands used in this study cover the spectral range between 420 and 1000 nm at a spectral sampling interval of 6.5 nm and a spectral bandwidth of 8.1 nm. The VNIR bands maintain a signal-to-noise ratio (SNR) exceeding 400:1 and spectral stability better than 0.5 nm thanks to the instrument’s onboard calibration assembly^45^. These attributes render the EnMAP data an excellent choice for remote sensing mapping of REEs.

### Processing methodology

We applied a curve-fitting technique using a 4th-order polynomial^46^ to detect and map REEs within the L2A data product. This technique enabled us to characterize the main absorption features of Nd at ∼580, ∼740, ∼800, and ∼865 nm (depicted in Fig. 1), as well as the carbonate feature between 2330 to 2340 nm. To achieve this, the local continuum was first removed between 520 to 900 nm for the VNIR and between 2230 to 2400 nm for the SWIR bands. Then, separate polynomials were fitted to the continuum-removed spectra within the ranges of 720–778, 770–825, 565–605, 825–895, and 2305–2365 nm (Fig. 1). Subsequently, the (real) root of the explicit first derivative was used to determine the wavelength of minimum reflectance (minimum wavelength). The coefficients of the fitted polynomial were also used to retrieve the depth, area, and width of the diagnostic absorption features. To eliminate the interfering effects of O_2_, the EnMAP band corresponding to oxygen’s residual absorption feature at 764 nm (band 62) was omitted from the calculations.

The retrieved spectral parameters then were subsequently employed in a stepwise decision-making process to identify Nd-bearing pixels. Initially, the pixels meeting the following criteria were isolated:

1 $$\left\{\begin{array}{l}735<740\,W<755 \,nm\\ 793<800\,W<805\, nm\\ 0.02<740\,D\, and \,800\,D\end{array}\right.$$

where λW and λD are the minimum wavelength and depth of the absorption feature centered at wavelength λ (nm). These results were further refined by retaining only the pixels that were linearly aligned in the scatterplot of 740D against 800D. Subsequently, the area of the 800 nm feature was used to represent the relative abundance of Nd in the mapped pixels. The relative abundance of carbonate rocks in the area was mapped based on the carbonate feature at ∼2340 nm (D > 0.13). The distribution of iron oxides was also mapped using $\frac{{\lambda }_{690(nm)}}{{\lambda }_{450(nm)}}>$ 2.5. Finally, to better understand the spectral and statistical variability of the mapped pixels, 2D scatterplots were prepared from the retrieved spectral parameters. All these processes were applied to a spatial subset of the mosaicked EnMAP data covering the Sulphide Queen mine and the surrounding areas.

The obtained results were validated in three ways: (i) by comparing the EnMAP spectra to laboratory-based spectral measurements of a hand specimen collected from the Sulphide Queen mine, (ii) by superimposing the yielded anomalies over high-resolution satellite images of the area, available on Google Earth, and (iii) by matching the anomalies with local geologic data. The reflectance spectral data was collated from the datasets published by Neave, et al.^9^. The corresponding specimen (CR36), containing 30,848 ppm (∼3%) Nd, has been measured using an ASD Field-Spec Pro FR spectroradiometer, with sampling intervals of 1.4 and 2 nm between 350–1000 and 1000–2500 nm, respectively. The final spectrum has resulted from averaging tens of evenly spaced repeat measurements taken from across the sample surface so that the 1σ of the spectrum was < 0.5% relative^9^.

## Data Availability

All EnMAP data are freely available through the EnMAP data access portal at the following link: https://www.enmap.org/data_access/. The EnMAP data are licensed products of DLR [2022], all rights reserved.
